# Rotavirus Burden, Genetic Diversity and Impact of Vaccine in Children under Five in Tanzania

**DOI:** 10.3390/pathogens8040210

**Published:** 2019-10-29

**Authors:** Joseph J. Malakalinga, Gerald Misinzo, George M. Msalya, Rudovick R. Kazwala

**Affiliations:** 1 Food and Microbiology Laboratory, Tanzania Bureau of Standards, Ubungo Area, Morogoro Road/Sam Nujoma Road, P.O. Box 9524, Dar es Salaam, Tanzania; joseph.malakalinga@sacids.org; 2 Southern African Centre for Infectious Disease Surveillance (SACIDS), Africa Centre of Excellence for Infectious Diseases of Humans and Animals in Eastern and Southern Africa (ACE), Sokoine University of Agriculture (SUA), P.O. Box 3297, Chuo Kikuu, SUA, Morogoro, Tanzania; gerald.misinzo@sacids.org; 3 Department of Animal, Aquaculture and Range Sciences, College of Agriculture, Sokoine University of Agriculture, P.O. Box 3004, Morogoro, Tanzania; msalya@sua.ac.tz; 4 Department of Veterinary Medicine and Public Health, College of Veterinary Medicine and Biomedical Sciences, Sokoine University of Agriculture, P.O. Box 3021, Morogoro, Tanzania

**Keywords:** rotavirus, genetic diversity, diarrhoea, vaccine effectiveness, Tanzania

## Abstract

In Tanzania, rotavirus infections are responsible for 72% of diarrhea deaths in children under five. The Rotarix vaccine was introduced in early 2013 to mitigate rotavirus infections. Understanding the disease burden and virus genotype trends over time is important for assessing the impact of rotavirus vaccine in Tanzania. When assessing the data for this review, we found that deaths of children under five declined after vaccine introduction, from 8171/11,391 (72% of diarrhea deaths) in 2008 to 2552/7087 (36% of diarrhea deaths) in 2013. Prior to vaccination, the prevalence of rotavirus infections in children under five was 18.1–43.4%, 9.8–51%, and 29–41% in Dar es Salaam, Mwanza and Tanga, respectively, and after the introduction of vaccines, these percentages declined to 17.4–23.5%, 16–19%, and 10–29%, respectively. Rotaviruses in Tanzania are highly diverse, and include genotypes of animal origin in children under five. Of the genotypes, 10%, 28%, and 7% of the strains are untypable in Dar es Salaam, Tanga, and Zanzibar, respectively. Mixed rotavirus genotype infection accounts for 31%, 29%, and 12% of genotypes in Mwanza, Tanga and Zanzibar, respectively. The vaccine effectiveness ranges between 53% and 75% in Mwanza, Manyara and Zanzibar. Rotavirus vaccination has successfully reduced the rotavirus burden in Tanzania; however, further studies are needed to better understand the relationship between the wildtype strain and the vaccine strain as well as the zoonotic potential of rotavirus in the post-vaccine era.

## 1. Introduction

Rotavirus group A (RVA) is the major causative agent of diarrhea. A recent analysis at the global, regional, and national levels using a standard year (2013) and a standard list of 186 countries revealed that in children under 5 years of age, rotavirus deaths ranged from 197,000 to 233,000, with almost half of these deaths occurring in sub-Saharan Africa [1,2]. Rotavirus infection can be controlled by improved sanitation, good hygiene, and vaccination, with vaccination being the most promising control method [1,3,4]. Rotavirus is a double-stranded RNA (dsRNA) virus belonging to the family *Reoviridae*. The genome consists of 11 segments of dsRNA. Six of the segments encode for six structural viral proteins (*VP*), namely, *VP1*, *VP2*, *VP3*, *VP4*, *VP6*, and *VP7* [5]. The remaining five segments encode for six non-structural proteins (*NSPs*), namely, *NSP1*, *NSP2*, *NSP3*, *NSP4*, *NSP5*, and *NSP6* [5]. *VP4* and *VP7* play a role in genotype-specific induced immunity and are targets for vaccine development and production [6,7,8]. It is believed that accumulated point mutations in the segments encoding for *VP4* and *VP7* are associated with the acquisition of different or novel antigenic properties that help the rotavirus strain to escape host neutralization antibodies induced by the vaccine and lead to the generation of virus diversification [9,10]. Similarly, the presence of glycosylation sites in the RotaTeq vaccine *VP7* at amino acid residue 238 is associated with a reduction in immunogenicity of the 7-1a epitope [10]. The glycosylation of amino acid residue 238 has also been reported to reduce the neutralization of animal RVA by monoclonal antibodies and hyper-immune sera [10,11,12]. Therefore, monitoring changes in circulating rotavirus strains over time is an essential method for assessing vaccine effectiveness. Furthermore, rotavirus is categorized into six groups, A to H, based on the *VP6* gene nucleotide sequence classification [13]. Groups A to C have been shown to infect both humans and animals [5,13,14]. Members of Rotavirus Group A are classified according to their glycoprotein (G) structures, namely, G (G1, G2, G3, …, Gn) genotypes, and their protein cleavage (P), namely, P (P[1], P[2], P[3], …, P[n]) genotypes [5,15]. Currently, 36 G genotypes and 51 P genotypes have been identified in humans and animals worldwide [16]. Globally, the most common G and P genotype combinations include G1P[8], G2P[4], G3P[8], G4P[8], and G9P[8], with G1P[8] being the most prevalent [17,18,19]. In Africa, the most common rotavirus genotype combinations detected between 2006 and 2015 were G1P[8], G2P[4], G9P[8], G2P[6], G12P[8], and G3P[6], with G1P[8] and G2P[4] being the most dominant [20,21,22]. Unusual genotypes included G1P[4], G2P[8], G9P[4], G12P[4], G8P[6], G8P[8], G12P[6], and G12P[8] [20,21,22]. This degree of diversity in rotavirus strains may have implications for vaccine effectiveness; thus, continuous genotype monitoring is important for monitoring vaccine impact, improvement, and development. Recently, a new classification system was developed by the Rotavirus Classification Working Group (RCWG), which involves the sequencing of all 11 RNA segments (whole-genome sequencing) and locating genotypes based on the percentage nucleotide sequence identity cutoff value from each segment (Gx–Px–Ix–Rx–Cx–Mx–Ax–Nx–Tx–Ex–Hx, where x stands for numbers such as 1, 2, 3, …, n) [23]. Along with phylogenetic analysis, whole-genome sequencing provides broad viral factor information, such as regarding origin, evolutionary relationships, interspecies transmission, antigenic shift (reassortment), antigenic drift (accumulated point mutation), and gene rearrangements [7,23]. All of these events contribute to the genetic diversity of the human rotavirus, which leads to reduced vaccine effectiveness [10,15,21,24]. Therefore, an understanding of genetic diversity within the country after vaccine introduction is necessary for the design of effective control programs. 

Two rotavirus vaccines have been internationally licensed, Rotarix (GlaxoSmithKline Biologicals, Rue de l’Institut, Rixensart, Belgium) and RotaTeq (Merck and Co., Inc., Kenilworth, NJ, USA). Both vaccines were found to be efficacious and safe, with high efficacy (range: 85–100%) in developed countries [25,26] and moderate efficacy (range: 39.1–61.2%) in developing countries [27,28]. By 2017, rotavirus vaccines had been introduced in 92 countries worldwide, with 32 of those countries being in Africa [29]. Despite the moderate vaccine efficacy in sub-Saharan Africa, it is expected that a decline in efficacy will result from changes in rotavirus strain patterns after vaccine introduction [30]. To evaluate vaccine performance, it is important to assess the disease burden and rotavirus genotype trends both before and after the introduction of vaccination to a country. This is necessary in order to assess vaccine performance and to aid decision making in areas such as the need to extend coverage for a particular vaccine strain, the replacement of vaccines, or the need for development of new vaccines that can provide effective disease control. 

In Tanzania, the Rotarix vaccine (RV1) was introduced in 2012 and implemented in the national immunization program in January 2013 [21,31]. The level of vaccine coverage was 85% 97%, 98%, 96%, and 100% in 2013, 2014, 2015, 2016, and 2017, respectively [32]. Several studies were conducted to determine the prevalence and genetic diversity of rotavirus in children under five before and after rotavirus vaccine introduction [20,21,22,31,33,34,35,36,37,38,39,40,41,42,43,44]. The purpose of this review article is to elucidate the genetic diversity of rotavirus, potential changes in strain type and the change in rotavirus burden after the introduction of the rotavirus vaccine in Tanzania. 

## 2. Trends of Rotavirus Infection and Impact of the Rotavirus Vaccine in Tanzania 

**Figure 1:**
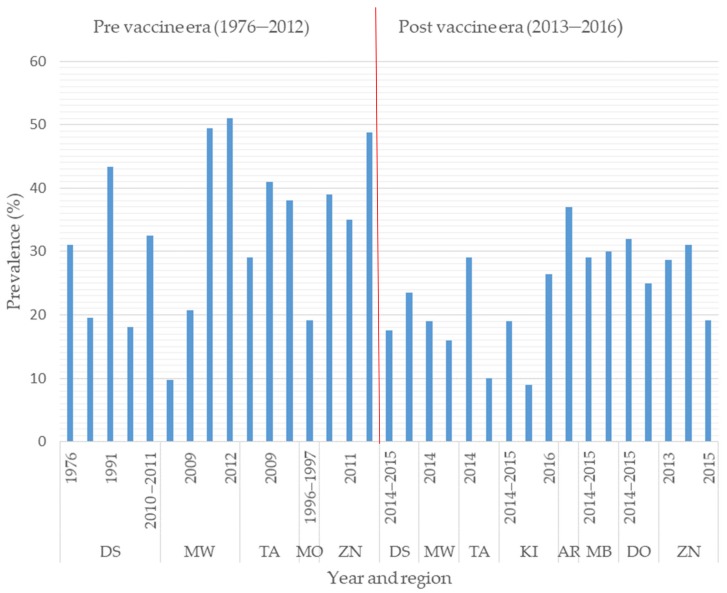
Histogram presentation of regional studies showing the rotavirus prevalence in children under five with diarrhea in Tanzania in pre vaccine era (1976–2012) and post vaccine era (2013–2015) (DS = Dar es Salaam, MW = Mwanza, TA = Tanga, MO = Morogoro, ZN = Zanzibar, KI = Kilimanjaro, AR = Arusha, MB = Mbeya).

In Tanzania, diarrhea is responsible for 9% (11,391/113,471) of deaths in children under five [28,45]. In 2008, rotavirus alone accounted for one-third of diarrhea-related hospitalization and over 72% (8171/11,391) of diarrhea-related deaths in children under five in Tanzania [28,45,46]. The prevalence of RVA infection based on the detection of the RVA antigen in fecal samples from children under five in Tanzania has been reported to range from 9.5% to 51% (Figure 1), with most cases occurring in children under two years of age [20,33,34,35,36,40,42]. A study in Mwanza in 2009 showed that children with rotavirus infection had prolonged hospital stays of 3.66 days compared to 2.5 days for children without rotavirus infection [42]. Prolonged hospital stays have an economic impact on parents in terms of the cost of treatment and the time spent at hospital. To mitigate the rotavirus infection burden, Rotarix vaccine was incorporated into the national vaccination program of Tanzania in January 2013 [31]. In the first year after introduction, the number of rotavirus-related deaths declined from 8171 to 2552 in children aged under five [4]. The prevalence of rotavirus infections in children aged under five with diarrhea ranged from 9.8% to 51% before vaccine introduction, and this declined to 9–37% after vaccine introduction, showing the positive impact of the vaccine. However, other factors such as improved sanitation and hygiene may also have contributed to this decline of prevalence. Based on an enzyme immunoassay (EIA) for the detection of RVA in fecal samples, a hospital-based study found a reduction in the prevalence of RVA infection in children under five years of age from 58% pre-vaccination to 18% post-vaccination in the Mwanza region and from 37% to 16% in the Tanga region of Tanzania [37]. The same study reported a reduction in the prevalence of RVA infection in the post-vaccination period from 26% in 2014 to 18% in 2015 in Mbeya [37]. In Dar es Salaam, based on EIA for the detection of RVA in fecal samples, the prevalence of RVA in children under five declined from 18.1–43.4% in the pre-vaccination period to 17–23.5% during the post-vaccination period (Figure 1), whereas the prevalence of RVA in Mwanza declined from 9.8–51% [36,37,43] to 16–19% [37]. Similar findings were observed in Tanga, where the prevalence of RVA declined from 29–41% [20,37] to 10–29% [37]. These results are complemented by other similar case control studies conducted in the post-vaccination era, which found a 44.9% reduction in rotavirus hospitalization in Manyara in 2015 [47] and reductions of 40%, 46%, and 69% in Zanzibar in 2013, 2014, and 2015, respectively [33]. The progressive reduction in rotavirus hospitalizations in Zanzibar, Mbeya, Dodoma and Moshi could be attributed to the increasing coverage of the rotavirus vaccine. This suggests that vaccine performance can be improved by maximizing coverage, both in the study regions and elsewhere in Tanzania. No pre- and post-vaccination data are available for the other 24 regions and the impact of vaccine introduction is therefore unknown in these regions. Hence, further studies with the aim of assessing the impact of vaccine introduction in these regions are necessary, as most of the regions are geographically and seasonally different. The findings from previous studies show that the Rotarix vaccine has had a positive impact on the reduction of rotavirus deaths, virus positivity and hospitalizations due to diarrhea. Similarly, a reduction in the rotavirus burden has been reported in neighboring countries such as Malawi [48,49] and Rwanda [50].

**Figure 2:**
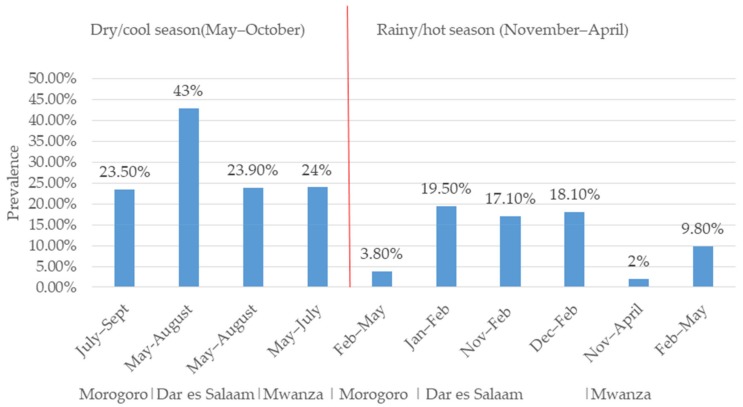
Rotavirus prevalence variation in children under five in Tanzania during dry/cool and rainy/hot season.

The difference in RVA prevalence between studies may be explained by seasonal variation. We observed a higher prevalence of RVA in studies conducted during the cool/dry season than those conducted in the hot/rainy season (Figure 2). These findings suggest that the rotavirus prevalence in the tropics is higher during the cool/dry season compared to the hot/rainy season [51]. For example, the prevalence of RVA based on the latex agglutination test (LAT) for the detection of the RVA antigen from children’s faeces in Morogoro was 23.5% (82/348) in the dry/cool season (July–September) and 3.8% (4/103) in the rainy/hot season (February–May) (Figure 2). A similar finding was observed in Dar es Salaam, where a higher prevalence of RVA was found in studies conducted in the cool/dry season (43%, n = 99, May and August) [41] than in the hot/rainy season (19.5%, n = 89, January–February) based on the LAT [39] (Figure 2). In the same region, based on EIA, the prevalence of RVA was found to be higher in the dry/cool season (23.9%, May–August) [31] than in the rainy/hot season (17.1%, November–February [31]; 18.1%, n = 270, December–February [40]) (Figure 2). In Mwanza, based on EIA, a higher prevalence (29%, n = 197) of RVA was observed in the dry/cool season (May–July) and a lower prevalence (0–2%, n = 197) was observed in the rainy/hot season (November–April) [37]. In same region, the rainy/hot season (February–May) was shown to have a lower prevalence of RVA 9.8% (n = 805) based on EIA [43]. However, in the same Mwanza region, a higher prevalence of 50.2% was observed in the rainy season (November–April) compared with 44.6% in the dry season (May–October), although the difference was not statistically significant [36]. In Tanga, a higher prevalence was observed in the dry/cool season (May and October) compared with the rainy/hot season (November–April) in 2009, 2014, and 2015 both before and after vaccine introduction, even though no seasonal variation was observed in 2011 [37]. Generally, the prevalence of RVA seems to be higher during the dry/cool season in the study regions (Figure 2). 

**Figure 3:**
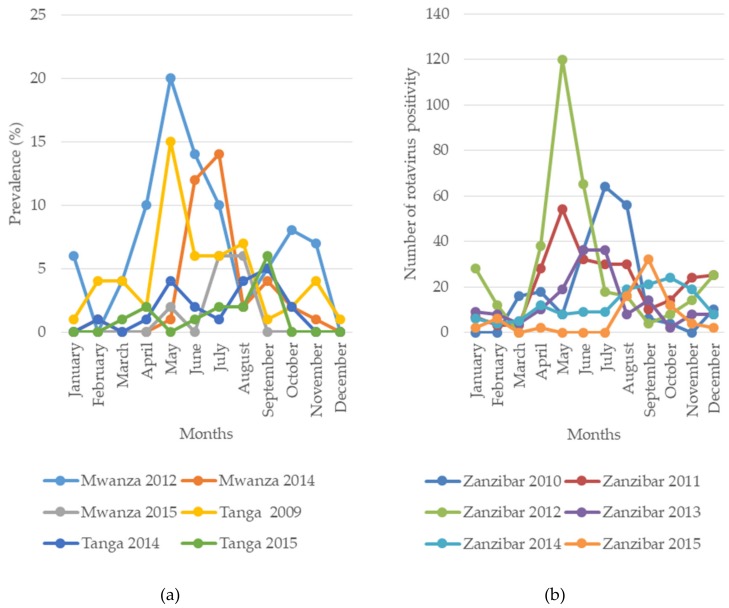
Seasonal pattern and peak months of rotavirus positivity before (2009–2012) and after (2014–2015) vaccine introduction in mainland (**a**) Tanzania and (**b**) Zanzibar.

The impact of vaccine introduction on seasonal peaks of RVA cases has been clearly observed in Mwanza, Tanga, Zanzibar and Manyara, with a seasonal peak delay of 1–4 months observed after vaccine introduction (Figure 3). Similar findings in the delay of the seasonal peak after vaccine introduction have been reported in the United States [52]. Before vaccine introduction, the peak months were May 2009 and August 2011 in Tanga, May 2012 in Mwanza, May–July of 2010–2012 in Manyara and July 2010 and May of 2011–2012 in Zanzibar [33,37,47]. After vaccine introduction, the peaks in rotavirus cases were delayed for 1–4 months towards the end of the year; in Tanga, the peak was in September of 2014–2015; in Mwanza, it was in July 2014 and July and August of 2015; in Manyara, it was in August–October of 2013–2015 and in Zanzibar, it was in June and July of 2013, September 2014 and October 2015 [33,37]. This information on the changes in seasonal peaks after vaccine introduction is crucial for the timing of rotavirus investigations. However, continual surveillance is necessary across the country to confirm these changes.

In addition, based on EIA, studies in Mwanza, Manyara and Zanzibar have shown that the vaccine effectiveness (VE) against rotavirus hospitalization in Tanzania ranges from 53% to 75%, with an average effectiveness of 61% for two doses of Rotarix vaccine (RV1) in children aged 5–23 months of age (Table 1). This is a moderate level of vaccine effectiveness; therefore, having a way to improve vaccine effectiveness could improve the performance of the vaccine in terms of reducing the number of rotavirus diarrhea hospitalizations and deaths. The performance of the vaccine may also be improved by increasing the vaccine strain coverage, developing a new, more effective vaccine and the transmission of animal strain to humans could be avoided by carrying out animal vaccination, which is not currently practiced in Tanzania. Other ways to improve vaccine performance include the use of zinc supplementation or the antisecretory agent racecadotril, adjusting the duration of breastfeeding, improving nutrition, and carrying out alternate dosing schedules [30]. The VE has only been determined in two (Mwanza and Manyara) out of the 25 regions in mainland Tanzania, as well as in Zanzibar. Mwanza and Manyara are found in northern Tanzania, while Zanzibar is an island in eastern Tanzania [53]. These regions represent the northern part of mainland Tanzania; therefore, further studies across the country are necessary to generate generalizable findings on vaccine effectiveness to allow for accurate decision making. The vaccine effectiveness in Tanzania is similar to that of bordering and other African countries, with a VE of 58.3% in Malawi [48], 56% in Zambia [54], 54% in Botswana [55], and 57% in South Africa [56]. The moderate vaccine effectiveness observed in Tanzania and other bordering countries may be attributed to the wide rotavirus strain diversity in Africa, infection with multiple genotypes at once, the high rate of mutation in the rotavirus genome, and the close proximity of inhabitants to animals [57,58]. This highlights the need to understand the shared and evolutionary relationships among rotaviruses circulating between humans and animals in most African countries, including Tanzania, where little is known regarding rotavirus infection in animals and humans, particularly in rural areas where inhabitants live in close proximity to animals. In addition, rotavirus can be carried asymptomatically [59], and 7.7–15% of children in Dar es Salaam were found to be carriers [41]. Asymptomatic carriers of rotavirus may act as reservoirs for the infection of susceptible individuals [40,42]. Therefore, the role of asymptomatic individuals in the propagation of rotavirus infection needs to be investigated in Tanzania.

Overall, studies have shown that the Rotarix vaccine has successfully reduced the number of rotavirus diarrhea cases, hospitalizations and deaths and has changed the seasonal patterns of rotavirus in the studied regions in Tanzania. However, further studies are needed across the country to form generalizable conclusions. Almost all available studies were hospital-based and located in urban and peri-urban areas. Hence, there is a need for more information from rural areas; future studies should therefore involve participants from communities and dispensary/health centers in rural areas. 

## 3. Trends in Rotavirus Genetic Diversity in Tanzania

In Tanzania, there is high diversity in RVA strains circulating among children under five years of age (Table 2). Studies from the years 2002–2018 reported circulating genotypes including G1P[8], G2P[4], G1P[6], G1P[4], G3P[8], G3P[6], G8P[4], G8P[6], G8P[8], G4P[4], G4P[6], G9P[8] and G12P[6], along with untypable and mixed strains (Table 2). The high observed strain diversity may be one of the reasons for vaccine effectiveness being only moderate in Tanzania. Strain diversity has been shown to hinder vaccine performance in terms of both effectiveness and efficacy in Africa [24]. In Dar es Salaam, Mwanza and Tanga, G1P[8] was the predominant genotype (24–75.1% of detected strains) both before and after vaccine introduction, followed by G2P[4] (8–50%) and G1P[6] (0.5–17%) (Table 2). G1P[8] is the most dominant of the detected genotypes, accounting for 24–64.7% of genotypes [20,21,22,31] before vaccine introduction and 41–75.1% after vaccine introduction [21]. Variation in the prevalence of rotavirus genotypes was observed in Dar es Salaam between 2005–2006 and 2010–2012, when G9P[8] accounted for 80% of the detected genotypes compared to 10% for G1P[8] in 2005–2006 [40]. A shift was observed in 2010 to 2011, when G1P[8] accounted for 64.7% of the detected genotypes compared to 0.5% from G9P[8] [31]. In another study, conducted in children aged under five years of age in Mwanza, Tanga, Mbeya, Dodoma, Kilimanjaro and Dar es Salaam, G2P[4] was shown to dominate, accounting for 50% of the genotypes compared to 15.63% G1P[8] and 15.63% G3P[6] [37]. Domination of G2P[4] over G1P[8] after vaccine introduction was reported in Tanga, Mwanza, Moshi, Mbeya, Dar es Salaam and Dodoma between 2009 and 2015 [48], which may have implications for vaccine effectiveness in these regions, as Rotarix vaccine is more efficacious against G1P[8] (68.3%) compared to the G2P[4] strain (49.3%) [60]. It is difficult to conclude whether G1P[8] is being suppressed in these regions due to the Rotarix vaccine, as G1P[8] is the main component of the vaccine cocktail. However, it is common for the rotavirus strain prevalence to fluctuate year after year—a certain genotype may dominate in one season and be completely absent in the next season [61,62,63]. The variation in rotavirus genotypes in Tanzania highlights the need for continuous monitoring of rotavirus genotypes to assess vaccine effectiveness, detect non-vaccine genotypes, and develop effective local multistrain vaccines. 

Despite the moderate vaccine effectiveness in Tanzania, very little is known about the relationship between the circulating strain and vaccine strains in Tanzania. Only one study prior to vaccine introduction sequenced the antigenic region (*VP7* and *VP4*), and a distant relationship was observed between the data on the vaccine strains in GenBank and the circulating strain in Dar es Salaam [31]. This provides a potential clue regarding the vaccine performance in the region, as the circulating strain might have been antigenically different to the vaccine strains. Due to the segmented genome structure of rotavirus, circulating strains may evolve rapidly through accumulating point mutations, reassortment, gene rearrangement, or due to vaccine pressure, thus driving an increase in rotavirus genetic diversity [10,15,21]. This kind of rotavirus evolution may lead to the emergence of new or novel strains that may escape neutralization antibodies [10,15]. Additionally, the parental strain of the Rotarix vaccine strain introduced in Tanzania was isolated 31 years ago in the United States [64]. Since RVA can accumulate point mutations in its antigenic regions, it is possible that the antigenic region of the circulating strain differs from those of the strains in the vaccine, thus allowing it to evade neutralization antibodies. This scenario is supported by the evidence of a moderate vaccine effectiveness of 54% in Mwanza and 53% in Zanzibar [21,33]. Also, there is evidence of vaccinated individuals shedding RVA strains in Arusha and Kilimanjaro [35,38]; however, it has not been determined whether the strains being shed are vaccine strains or circulating strains. Therefore, it is very important to ascertain the genetic relationship between circulating rotavirus strains from distinct geographical locations as compared with the strains in the vaccine used in Tanzania and elsewhere in this post-vaccine era. 

In addition, high percentages of untypable, mixed genotype infections, atypical/unusual (G1P[4]) and classical animal origin genotypes (such as G8P[6], G4P[6] and G3P[6]) have been detected in studies conducted in Zanzibar, Mwanza, Dar es Salaam, Mbeya, Dodoma and Tanga (Table 2). The occurrence of animal origin genotypes in humans may indicate direct interspecies transmission or the occurrence of reassortment events between human and animal rotaviruses in Tanzania, which needs to be further clarified. The existence of atypical/unusual G1P[4] genotypes is believed to have arisen through inter-genogroup or inter-genotype reassortment between Wa- and DS–1-like strains [21,65] and untypable RVA strains are also thought to have originated from animals [65,66,67]. Therefore, understanding the zoonotic potential of livestock RVA is of particular interest in Tanzania as, currently, the potential sharing of RVA genotypes between humans and animals remains unknown. In the rural areas of developing countries, including Tanzania, people live in close proximity to animals and sanitation, and hygiene may be poor, thus increasing the risk of zoonotic rotavirus infection [68]. Infected animals or humans shed over 10 billion infectious rotavirus particles per gram/milliliter stool [69], thus contaminating environmental objects. Rotaviruses are very stable in the environment and can withstand harsh conditions for several weeks on surfaces and water (portable and recreational water) [70]. Fecal–oral transmission can occur from person to person, or through contact with an infected animal, fecal–contaminated fomites, hands, food and water. Zoonotic rotavirus transfer may occur either by direct transmission from animals to humans or through the exchange of RNA segments, such as through reassortment [71,72]. Strains such as G6, G8, G5, G10, G11, P[1], P[2], P[3], P[5], P[7] and P[12] are commonly detected in animals, and G2, G3, G4, and G9, P[6], are common in both humans and animals [71]. The classic animal strains G8P[6], G4P[6] and G3P[6] have been detected in children under five in Tanzania [33,36,37,40]; however, the studies did not further investigate whether the infections were due to direct interspecies transmission or reassortment events or both. Direct transmission of rotaviruses from animals to humans has been suspected in children with acute diarrhea in Thailand (infected with G10P[14]), Italy (infected with G10P[14] and G8P[14]), Cameroon (infected with G5P[7]) and Nigeria (infected with G8P[1]) [73,74,75]. Reassortment between human and animal strains may increase the human-to-human transmission of the resultant reassortant strain [76]. Unusual reassortant strains, such as the G6P[8] strain, have been detected in Bangladesh [27].

The observed mixed genotype infections may facilitate reassortment events, which is one of the mechanisms for increasing rotavirus strain diversity [7,21,77]. Due to the segmented nature of rotaviruses, there is the possibility of segment exchange in mixed genotype infections. This kind of rotavirus evolution may generate reassortant strains with novel antigenic properties that are capable of fast spread in human populations [76]. Since there is evidence of untypable, mixed infection strains and strains of animal origin circulating in humans in Tanzania (Table 2), there is a need to understand the zoonotic potential and reassortment events of the circulating rotavirus strains to allow for the effective design and implementation of strategic intervention programs, including the reduction or limitation of strain diversity in human rotaviruses. Understanding the occurrences of these phenomena cannot be achieved by RT-PCR genotyping alone; rather, sequencing is needed of antigenic regions such as *VP4* and *VP7*, or whole-genome sequencing of selected strains including those of animal origin and unusual, untypable, and mixed genotype infection strains. Therefore, after PCR genotyping, sequencing may be important for tracking changes over time as the virus evolves [77,78], to enable comprehension of the rotavirus vaccine performance or effectiveness. 

Overall, there have been no major changes in rotavirus strain patterns. G1P[8] was predominant in the pre-vaccine period, and is still the predominant strain after vaccine introduction. However, continuous strain surveillance is crucial to allow for a clear understanding of the impact of the vaccine on RVA genotype patterns, as well as the early identification of emerging, unusual and novel genotypes not covered by the vaccine. Also, it would be beneficial for Tanzania and other African countries to develop their own vaccines based on the local circulating strains. 

## 4. Conclusions

The Rotarix vaccine successfully reduced the rotavirus burden in Tanzania; however, due to the moderate vaccine effectiveness, we recommend that further studies be carried out to determine the vaccine effectiveness across the country and to clarify the genetic and antigenic relationships between the circulating rotavirus strains and the strains in the vaccine. In Tanzania, very little is known about the genetic composition of circulating rotavirus strains, and we therefore suggest that whole-genome sequencing be performed on selected circulating rotavirus strains from distinct geographical locations in order to understand viral factors and mechanisms associated with rotavirus genetic diversity in Tanzania. As only a few studies have been conducted on rotavirus genotypes after vaccine introduction, further studies are needed in order to better understand the impact of the vaccine on rotavirus strain patterns and to identify novel, reassortant, unusual and untypable strains that are present in the post-vaccine era in Tanzania.

## Figures and Tables

**Table 1:** Rotarix vaccine effectiveness (case control studies) in Tanzanian regions in children aged 5–23 months and the impact of the vaccine on the reduction of hospitalizations.

Region	Reference	Diagnostic Technique	Settings, Year of Study	Rotavirus Vaccine Dosage Comparison	VE (95% CI)
Zanzibar	[1]	EIA	Single hospital, December 2012–2015	2 doses vs. 0 doses	57 (14–78)
Manyara	[47]	EIA	Single hospital, August–December 2015	2 doses vs. 0 doses 2 doses vs. 0 doses	74.8 (−8.2 to 94.1) 85.1 (26.5–97.0)
Mwanza	[28]	RT-PCR	Multiple hospitals, May 2015	2 doses vs. 0 doses ≥1 dose vs. 0 doses	49 (−30 to 80) 53 (−14 to 81)

EIA = enzyme immunoassay, RT-PCR = reverse transcription polymerase chain reaction.

**Table 2:** Regional rotavirus genotype distribution in Tanzania, showing the most to the least prevalent genotypes per given study.

Region	Ref	Year	n	G1P [8]	G2P [4]	G9P [8]	G12P [6]	G12P [8]	G1P [4]	G8P [4]	G8P [6]	G4P [6]	G4P [4]	G3P [6]	G3P [8]	G8P [8]	G1P [6]	Untypeable	Mixed genotypes
Dar es Salaam	[48]	2005–2006	49	10%	0	90.7%	0	0	0	0	0	0	0	0	0	0	5%	10%	0
	[47]	2010–2011	211	64.7%	0	0.5%	11.1%	0	3.7%	14.2%	3.2%	1.1%	1.1%	0	0	0	0.5%	0	0
Tanga	[49]	2007–2008	32	34%	0	3%	0	0	0	0	0	0	0	0	0	0	3%	28%	31%
Mwanza	[25]	2010–2011.	100	24%	2%	0	0	0	0	7%	4%	0	0	0	0	6%	17%	0	29%
Zanzibar	[1]	2010–2015	101	52%	8%	0	0	2%	0	0	3%	0	0	16%	0	0	1%	7%	12%
ND	[28]	2009–2015	32	15.63%	50%	0	0	0	3.1%	0	0	0	0	15.6%	3.1%	0	3.1%	6.3%	9.3%
ND	[62]	2010	–	31.4%	–	–	–	–	–	–	–	–	–	–	–	–	–	–	–
ND	[62]	2011	–	25.8%	–	–	–	–	–	–	–	–	–	–	–	–	–	–	–
ND	[62]	2014	–	41.1%	–	–	–	–	–	–	–	–	–	–	–	–	–	–	–
ND	[62]	2015	–	75.1%	–	–	–	–	–	–	–	–	–	–	–	–	–	–	–
East Africa	[63]	2007–2011	–	23%	8%	12%	0	4%	0	4%	5%	0	0	4%	0	0	4%	0	0

ND: Not determined to region level; “–“: Data unavailable.
